# Immune challenge affects risk sensitivity and locomotion in mosquitofish (*Gambusia holbrooki*)

**DOI:** 10.1098/rsos.241059

**Published:** 2024-10-30

**Authors:** Stella A. Encel, Ashley J. W. Ward

**Affiliations:** ^1^ School of Life and Environmental Sciences, University of Sydney, Camperdown 2006, Australia

**Keywords:** immune challenge, behaviour, sickness behaviour, lipopolysaccharide, risk, activity

## Abstract

The immune system is crucial in responding to disease-causing pathogens. However, immune responses may also cause stereotypical changes in behaviour known as sickness behaviours, which often include reduced activity. Sickness behaviours are thought to have an important role in conserving energy required to support the immune response; however, little is known about how they manifest over time or in relation to risk, particularly in fishes. Here, we induced an immune response in mosquitofish (*Gambusia holbrooki*) by inoculating them with *Escherichia coli* lipopolysaccharide (LPS). We subsequently tested batches of fish at 24 h intervals and examined: locomotory behaviour, tendency to use a refuge and fast-start response immediately following a threat stimulus (measured as peak acceleration). Control and LPS-treated fish behaved similarly on days 1, 3 and 4. However, 2 days post-inoculation, LPS fish swam more slowly and spent more time in the refuge than control fish, although no difference in post-threat peak acceleration was found. Our findings suggest that sickness behaviours peak roughly 2 days following exposure to LPS and are relatively short-lived. Specifically, immune-challenged individuals exhibit reduced locomotion and exploratory behaviour, becoming more risk averse overall while still retaining the ability to respond acutely to a threat stimulus.

## 1. Introduction

Pathogens, and the diseases that they cause, are known to affect the behaviour of host organisms [1,2]. However, many of these behavioural changes are driven not by direct effects of the pathogen itself but by the host’s immune response. Inflammatory immune responses are associated with stereotypical changes in behaviour such as lethargy, somnolence and anorexia [3–5]. In particular, these behaviours are most associated with a feature of innate immunity in vertebrates known as the acute-phase response [4,6,7]. The acute-phase response is a non-specific, transient systemic inflammatory reaction defined primarily by changes in the concentrations of a number of serum proteins known as acute-phase reactants, which include complement factors and pro-inflammatory cytokines [8,9]. These reactants cause sweeping alterations in cell-signalling patterns and leucocyte activity, which cumulatively constitute an organized physiological response to stimuli such as infection and injury [3,10,11].

The acute-phase response is highly conserved among vertebrates and generally follows a relatively predictable trajectory after an inflammatory stimulus [3]. Serum concentrations of many acute-phase reactants increase rapidly in this period, often peaking within 24–72 h and declining quickly thereafter, although some may take considerably longer to return to normal [8]. During this time, pro-inflammatory cytokines act directly on the brain to produce behavioural changes [12]. At the same time, the metabolic cost of mounting the immune response reduces the energy available for other activities [13–15]. Together, these changes in physiology give rise to a suite of responses known collectively as ‘sickness behaviours’. This broad term encompasses a diverse range of behaviours, often including depressed activity as well as reduced foraging, reproductive and social behaviour [16–18]. Many immunological studies have examined the acute-phase response by measuring concentrations of acute-phase proteins and other humoral immune factors over time [3,8]. However, less is understood about the extent to which the expression of sickness behaviours mirrors the trajectory of the acute-phase response, and detailed time series of these important behavioural changes are lacking.

Sickness behaviours are thought to be underpinned, at least in part, by the existence of an energetic trade-off between immune defence and other life-history traits. It is generally held that the physiological demands of an immune response require energy to be diverted from other functions such as growth, reproduction and general activity [19]. For instance, immune challenge has been shown to reduce activity in white-crowned sparrows (*Zonotrichia leucophrys gambelii*) [6], limit parental behaviour in mice (*Mus musculus*) [20] and decrease both courtship and mating behaviour in male guppies (*Poecilia reticulata*) as well as altering social behaviour in female guppies [21,22]. In addition to these effects, immune-challenged individuals may also have a diminished ability to respond effectively to cues that signal a potential threat, such as those related to predator activity. This, in theory, means that immune-challenged individuals may benefit by adopting more risk-averse patterns of behaviour during the course of their illness. While it is thought that sickness behaviours are an adaptive response to disease, promoting recovery through the conservation of metabolic resources [1,7] (but see [18]), less is known about how sickness behaviours manifest in relation to predation threat.

Alongside disease, predation is among the most persistent threats to the survival of most animals. Accordingly, prey are generally highly responsive to cues indicating predator proximity and activity [23,24], using this information to regulate risk-sensitive behaviours. Various factors may influence risk sensitivity in a prey animal including familiarity with the immediate environment, as well as the availability of refugia [25]. Specifically, many animals adopt a risk-averse approach when in novel environments or when access to refugia is restricted [26]. Intrinsic factors affecting an individual’s risk sensitivity include their sex, age and body size as well as more labile considerations such as physiological state [27–29]. For instance, satiated animals generally tend to be more risk-averse than their hungry counterparts [25]. While elevated immune activity is known to influence many aspects of an individual’s physiological state, less is known about how it affects behavioural responses to risk. House finches (*Haemorhous mexicanus*) infected with a bacterial pathogen were found to be less responsive to predator stimuli, an effect that has been ascribed to the locomotory depression caused by immune challenge [30]. In a wild study, Eurasian collared doves (*Streptopelia decaocto*) inoculated with lipopolysaccharide (LPS) as nestlings showed lower survivorship after fledging, with the main identified cause of this mortality being higher rates of predation [31]. These findings demonstrate that immune activity can have significant effects on responses to predation risk, which warrant further investigation.

The acute-phase response has been the subject of considerable scientific interest for almost a century (see [32]). Meanwhile, although sickness behaviours have also been recognized for several decades, they have not been examined in detail until comparatively recently. Sickness behaviours are now increasingly recognized as a crucial component of an organized systemic response to immune challenge rather than merely an inevitable or undesirable side-effect of immune activation [4,11,33]. In spite of this, attempts to establish a detailed time series that clarifies the extent to which the expression of sickness behaviours aligns temporally with the acute-phase response have been lacking. Additionally, although sickness behaviours are thought to be broadly consistent among vertebrates, very little research (apart from a small number of recent studies [17,34]) has focused on the manifestation of sickness behaviours in fishes [4] despite the fact that teleosts alone constitute the largest taxonomic group of vertebrates. This presents a concerning limitation in our ability to make generalizations about the nature and importance of behavioural responses to immune challenge in vertebrates. Furthermore, given the intense selective pressure that both disease-causing pathogens and predation risk exert on prey animals, the interaction between these two threats requires detailed investigation. Here, we examine the effect of immune challenge on locomotion, refuge use and response to a threat stimulus in mosquitofish (*Gambusia holbrooki*) across a 4 day (96 h) period.

## 2. Methods

### 2.1. Study species

Mosquitofish (*Gambusia holbrooki*) measuring 20.4 ± 4.9 mm (mean ± s.d.) were collected for use in the experiments in May 2023 from Manly Dam, Sydney (−33.806360 S, 151.235144 E). The mosquitofish is a small species of fish that inhabits shallow freshwater habitats and is preyed upon by a wide variety of predators, including fish, birds and insects [35]. Prior to experiments, the fish were held for one week in 180 l vats held at a temperature of 23.1 ± 1°C and a 12 : 12 light : dark cycle in the animal holding rooms at the University of Sydney. Fish were fed daily ad libitum with commercially available fish flakes (Nutrafin).

### 2.2. Immune challenge

Fish were randomly assigned to either LPS or control treatments (LPS-treated: *n* = 64, Control: *n* = 64). Groups of eight individuals were placed in a 500 ml bath of either LPS solution or aged tap water and left for 60 min before being transferred in their groups to separate 5 l holding aquaria. Fish assigned to the LPS treatment were bathed in a solution of aged tap water and LPS at a concentration of 100 mg l^−1^ (Sigma-Aldrich; lipopolysaccharides from *Escherichia coli* serotype O111:B4). This concentration was selected based on previous studies that induced immune reactions in fish using similar dosages [21,36,37]. LPS offers a valuable means of inducing an inflammatory response in vertebrates and has been widely used on subjects from various taxa for this purpose [6,38,39]. The use of LPS allows the effects of innate immune responses to be decoupled from the variable and disease-specific effects of live pathogens, allowing broader inferences to be made [40]. Following treatment, fish were monitored for signs of stress prior to experiments. Previous studies show that the immune response of mosquitofish peaks at approximately 48 h post-exposure [30]. The testing schedule (24, 48, 72 and 96 h) was chosen on this basis. The bath method was chosen over an intraperitoneal or intramuscular injection due to the small size of the fish and the success of this method in other trials using small teleosts [21,37,41].

### 2.3. Experimental protocol

A single fish was netted from its holding tank and introduced to an adjacent circular test arena, which had a diameter of 64 cm, and was filled to a depth of 8 cm with aged tap water at the same temperature as the holding tank (24°C). The arena was constructed from white Perspex. A single artificial aquarium plant was added to the arena. This had a circular base of diameter 3 cm, spreading to a diameter of 11 cm at the water’s surface. The plant was positioned 23 cm from the arena centre. The purpose of this was to provide a refuge for the fish. The arena was surrounded by white screens to minimize external disturbance and was lit by 2 × 20 W cool white LED strips. Following its introduction, the fish was allowed to acclimate to the arena for 10 min, after which we filmed for 5 min, using a Canon GX camera set into a recess in the ceiling of the screens, 1.2 m above the arena and filming at 24 fps at a resolution of 1920 px. Halfway through the filming process (i.e. 12.5 min after introduction and 2.5 min after we began filming), we dropped a 3 g cubic lead weight into the centre of the arena. The purpose of this was to simulate an attack by an aerial predator. At the completion of 5 min filming, the fish was removed and transferred to a new holding tank. We conducted tests on each of the 4 days, respectively 24, 48, 72 and 96 h following control treatment or inoculation with LPS. On each day, we tested 32 fish, comprising 16 LPS-treated fish and 16 control fish. All fish were tested between 14.00 and 16.00. Each test was done with a naive fish. Each fish was used only once. After testing, the sex and body length of the fish were recorded. Sex was determined by the presence or absence of a gonopodium (present in males and absent in females). Body size was measured with a ruler directly after testing.

### 2.4. Data extraction

Each video was tracked using TRex software [42], yielding a total of 7200 *x*, *y* coordinates for each fish (300 s at 24 fps). From this, we were able to calculate our three response variables, which were speed, time in the refuge and post-threat peak acceleration. We defined time in the refuge as being when a fish was within a radius of 5.5 cm of the centre of the artificial plant. We calculated speed based only on the time the fish spent outside the refuge. For the purpose of our analysis, we divided the trial into two halves: before the introduction of the lead weight (ante-threat) and after the introduction of the lead weight (post-threat). The delineation between the two was determined as the point at which the lead weight first made contact with the water surface. Acceleration was measured by examining the trajectories of fish in the 10 s immediately following the threat stimulus. We took the 10 highest values for acceleration (i.e. peak acceleration) in this time and calculated the median of these for each individual.

### 2.5. Statistical analysis

Statistical analysis was performed using R [43], and using packages lme4 [44], car, MuMIn [45] and ggplot [46]. We used the model.sel function in MuMIn to identify the most appropriate model for the error distributions in both cases. We subsequently used a generalized linear mixed model, specifying a gamma distribution, to analyse time in the refuge, accounting for the fact that this response variable was non-normal and right skewed. We used a general linear mixed model to analyse speed. Neither sex nor size was statistically significant (*p* > 0.8 in all cases), and so both were excluded from the final models. In each of our two models (‘Time in Refuge’ and ‘Mean Speed’), we specified three fixed effects: Treatment (Control or LPS), Day (1, 2, 3 or 4) and Threat (Ante-threat and Post-threat). We specified Fish ID as our random effect, to account for the repeated measures nature of the data in respect of quantifying behaviour before and after the threat stimulus. We used a general linear model to analyse acceleration, with Day and Treatment as our fixed factors, since there was only one measurement per fish.

## 3. Results

### 3.1. Time in refuge

**Figure 1.**
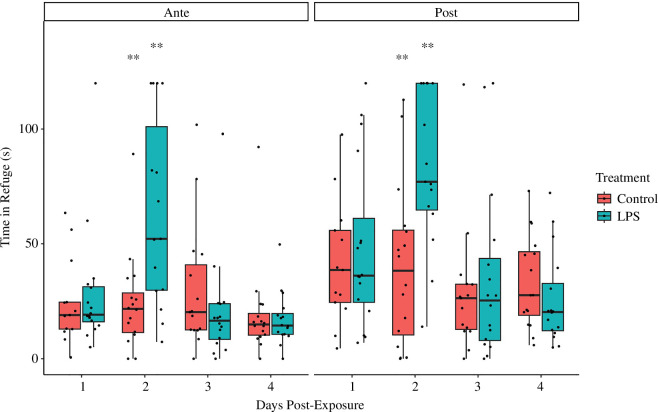
Boxplot showing mean time spent in the refuge (in seconds) for LPS-treated and control fish before and after a threat stimulus, tested over 4 days. Fish tested on day 2 spent significantly more time in the refuge than control fish both before and after the threat stimulus.

There was a significant interaction between Treatment and Day (table 1). LPS-treated fish spent more time in the refuge than control fish two days following inoculation, but no difference between treatments was apparent on any other day. Fish reacted strongly to the threat stimulus, spending more time in the refuge following the simulated predation event (figure 1), but there was no interaction of Threat with Treatment (table 1). Pairwise comparisons confirmed that LPS-treated fish spent significantly more time in the refuge than control fish at 2 days post-inoculation both before and after the threat stimulus but not on any other day (table 2).

**Table 1.** Output from GLMER showing main and interactive effects of LPS on mean time spent in refuge. Significance is denoted by asterisks (*p < 0.05; **p < 0.005).

factor	*χ*2	d.f.	*p* value
treatment	0.385	1	0.535
day	18.139	3	<0.001**
threat	15.108	1	<0.001**
treatment:day	10.5	3	0.015*
treatment:threat	0.007	1	0.933
day:threat	1.104	3	0.776
treatment:day:threat	2.291	3	0.514

**Table 2.** *Post hoc* pairwise comparisons of mean time spent in the refuge in LPS-treated and control fish. Significance is denoted by asterisks (*p < 0.05; **p < 0.005).

day	threat	estimate	s.e.	*z* value	*p* value
1	ante	−0.127	0.28	−0.454	0.65
2	ante	−0.991	0.27	−3.671	<0.001**
3	ante	0.451	0.27	1.671	0.095
4	ante	0.05	0.265	0.188	0.851
1	post	−0.131	0.28	−0.469	0.639
2	post	−0.788	0.27	−2.919	0.004**
3	post	−0.113	0.27	−0.418	0.676
4	post	0.269	0.265	1.013	0.311

### 3.2. Speed

**Figure 2.**
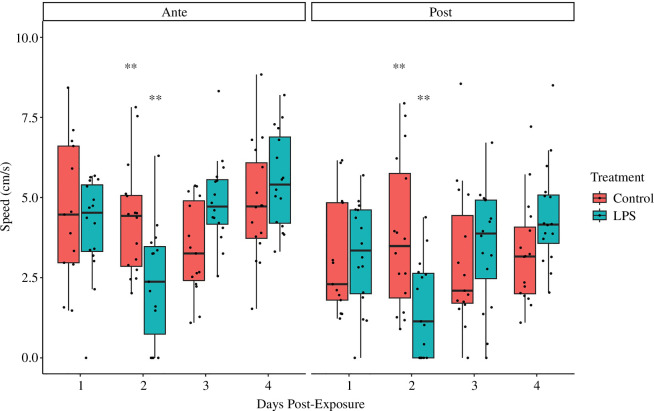
Boxplot showing mean swimming speed (in cm s^−1^) of LPS-treated and control fish before and after a threat stimulus. Fish were tested over 4 days. Fish tested on day 2 showed significantly slower mean swimming speeds than control fish both before and after the threat stimulus.

There was a significant interaction between Treatment and Day (table 3). LPS fish had slower swimming speeds than control fish 2 days following inoculation (figure 2). Fish reacted strongly to the threat stimulus, swimming more slowly following the simulated predation event, but there was no interaction with Treatment (table 3). Pairwise comparisons revealed that LPS-treated fish had significantly slower swimming speed than control fish at 2 days post-inoculation both before and after the threat stimulus but not on any other day (table 4).

**Table 3.** Output from GLMER showing main and interactive effects of LPS on mean swimming speed. Significance is denoted by asterisks (*p < 0.05; **p < 0.005).

factor	*χ* ^2^	d.f.	*p* value
treatment	0.023	1	0.88
day	6.838	3	0.077
threat	20.831	1	<0.001**
treatment:day	10.977	3	0.012*
treatment:threat	0.005	1	0.941
day:threat	3.769	3	0.288
treatment:day:threat	7.454	3	0.059

**Table 4.** *Post hoc* pairwise comparisons of mean swimming speeds in LPS-treated and control fish. Significance level is denoted by asterisks (*p < 0.05; **p < 0.005).

day	threat	estimate	s.e.	*z* value	*p* value
1	ante	0.501	0.661	0.758	0.985
2	ante	1.945	0.636	3.058	0.003**
3	ante	−1.489	0.636	−2.14	0.06
4	ante	−0.732	0.626	−1.169	0.848
1	post	−0.14	0.661	−0.212	0.832
2	post	2.251	0.636	3.66	<0.001**
3	post	−0.44	0.636	−0.691	0.491
4	post	−1.178	0.626	−1.883	0.061

### 3.3. Acceleration

There was no significant effect of either treatment or day with respect to acceleration (table 5).

**Table 5.** Model output from GLM showing main and interactive effects of treatment and day on median peak acceleration.

	sum sq.	d.f.	*F* value	*p* value
day	1.563	3	1.722	0.168
treatment	0.888	1	2.936	0.09
day:treatment	0.243	3	0.268	0.848

## 4. Discussion

In this study, all fish, irrespective of treatment or day, swam more slowly and spent more time in the refuge after the threat stimulus. This reflects a typical response to perceived risk [25]. Fish in the control treatment did not otherwise exhibit any significant changes in behaviour over the course of the study with respect to either speed or refuging behaviour. By contrast, fish inoculated with LPS demonstrated significant changes in both refuging and movement behaviour 2 days after inoculation. At this time, LPS-treated fish spent significantly more time in the refuge and had significantly lower swimming speeds than control fish. Acceleration in the 10 s immediately after the threat stimulus was also measured, but no significant differences were detected between control and LPS groups. LPS-treated and control fish demonstrated very similar refuging and swimming behaviour 1, 3 and 4 days post-inoculation. This suggests that the expression of sickness behaviours, including reduced activity and increased aversion to risk, peaks roughly 2 days post-exposure in mosquitofish. These findings are consistent with existing research in the same species that has shown that the metabolic effects of immune challenge with LPS do not become apparent until 2 days after inoculation [47].

The significance of this time period may be related to characteristics of the various cytokines that mediate sickness behaviours, which may be broadly categorized as either pro-inflammatory or anti-inflammatory. The concentrations of pro-inflammatory cytokines (e.g. IL-1β and TNF-α), which promote the manifestation of sickness behaviours, begin to increase almost immediately after initiation of the acute-phase response and may remain elevated for 12–72 h [12,48]. However, anti-inflammatory cytokines (e.g. IL-10), which downregulate the expression of sickness behaviours, activate more slowly and may not begin to increase in concentration for 24–72 h [48]. With this in mind, it may be that the peak activity of pro-inflammatory cytokines occurs roughly 2 days post-stimulus, after which their concentrations begin to decline as the production of anti-inflammatory cytokines rises. This being the case, it is logical that the most pronounced behavioural effects may be observed during this period. However, studies that directly measure these changes in cytokine concentrations alongside sickness behaviours are needed to elucidate this further. It has also been shown in other vertebrates that the duration of neuroinflammation caused by exposure to LPS is dose-dependent, with higher doses often producing longer lasting effects [49]. Since very few studies exist regarding the behavioural or neuroimmunological effects of LPS in fish, it is difficult to predict how dosage may affect their behaviour. Fish are sometimes said to possess more robust innate immune responses to endotoxin than other vertebrates due to their close association with Gram-negative bacteria in aquatic environments [50] and their less sophisticated adaptive immune systems [3]. However, the results of this study imply that sickness behaviours may be more consistent across vertebrate taxa than previously recognized. Broadly, the findings here are congruous with the existing state of knowledge about the acute-phase response and the accompanying manifestation of sickness behaviours [4], although a detailed time series of this has not been experimentally established until now.

Immune-challenged fish here showed a general reduction in activity 2 days after inoculation. It is broadly accepted that mounting an immune response is metabolically demanding [51,52] and often leads to reduced activity [17,53]. While this is typically attributed to differential allocation, allowing energy to be diverted from locomotion to support the immune response, there is still some uncertainty surrounding the adaptive value of reduced activity [18]. It has been shown that mosquitofish are able to compensate for the energetic demands of an immune challenge by upregulating ATP production in order to maintain comparable swimming speeds to control fish during a short swim test [47]. This supports our finding that LPS had no effect on peak acceleration after a threat stimulus, as it suggests that immune-challenged individuals can compensate physiologically to allow for acute bursts of necessary physical activity. Nonetheless, the same study found that immune-challenged fish lost body mass during the week-long experiment while control fish did not [47]. This implies that there is a meaningful trade-off between the immune response and other functions such as locomotion and maintenance of body condition as shown in other species [13,54,55]. Sickness behaviour is sometimes described as a change in motivational state, involving a reorganization of behavioural priorities which enables an animal to respond flexibly to the physical constraints of infection [56]. With this in mind, it may be that the adaptive value of reduced activity in immune-challenged individuals is that decreased motivation to explore and acquire resources minimizes the risk of exposure to competition, predation and indeed further contact with pathogens at a time when the energy available to respond to these threats is already diminished. This offers an interesting potential evolutionary explanation for some aspects of sickness behaviours but may be difficult to assess experimentally, in part because of likely interactions with other factors such as personality and sex. While no sex-based differences were found in this study, another study found that male *Psammodromus algirus* lizards had significantly lower sprint speeds after immune challenge, while females were unaffected [57]. Importantly, males had higher initial sprint speeds and immune-challenged males still equalled the speed of females, which implies that this change in escape response is unlikely to have a significant effect on predation risk for the males. While the complex and potentially antagonistic relationship between androgens and immune activity may be involved [58], it seems more likely in this case that males simply have a broader metabolic scope that accommodates greater plasticity [59] without compromising the effectiveness of predator avoidance. This aligns with previous assertions that since the potential fitness costs of predation generally exceed that of infection, behavioural responses to predation risk should override the manifestation of sickness behaviours [56]. Interestingly, a similar study of immune-challenged Iberian ribbed newts (*Pleurodeles waltl*) found no differences in swimming speed during a short-distance escape response measured 1 and 7 days after immune challenge [60]. While broadly consistent with the findings of this study, it is possible that the greater interval between testing precluded the ability to capture acute or short-lived changes in behaviour. This emphasizes the need for detailed time series of sickness behaviours to be established experimentally, as we attempt to do in this study.

Aside from infection, the greatest threat to the survival of most animals is predation. Consequently, animals have evolved predator aversion strategies alongside immune defences. Both predator avoidance and immune activity are dynamic traits in which individuals may vary their energetic investment at any given time. As such, there may be conflict between energetic investment in immune activity and predator defences, potentially driving a trade-off [61]. For instance, immune-challenged fish tested here demonstrated slower swimming speeds 2 days following exposure to LPS. One likely explanation for this is that the demands of the immune challenge divert energy from locomotion. Reduced swimming speed may compromise the effectiveness of an attempted escape response, making the immune-challenged individual more vulnerable to predation. However, we found no difference in peak acceleration between control and LPS-treated fish in the 10 s after the threat stimulus. Acceleration may be more important than mean speed in the immediate aftermath of an attack, as it more directly reflects the ability of the individual to initiate an effective flight response. This finding is consistent with aforementioned studies on escape responses [57,60] and with evidence showing that immune-challenged animals can, in some cases, upregulate their metabolism in the short term to support energetically demanding behaviours when needed [47]. However, reduced swimming speed would nonetheless be a significant disadvantage to the immune-challenged individual in the case of a protracted predator encounter or pursuit. Indeed, it is often said that individuals of inferior health status are preferentially targeted by predators [62], and it has been shown that animals mounting an immune response and those with lower immunocompetence may be more vulnerable to predation [63–65]. Interestingly, it has also been found that wild immune-challenged house sparrows (*Passer domesticus*) downregulate immune activity after being presented with a simulated predation threat [66]. This supports the idea that animals may navigate the trade-off between immune and predator defences by modulating their investment to reflect the relative risk posed by each at a given time. It also implies that animals may benefit by adopting more risk averse patterns of behaviour while they are mounting an immune response. Accordingly, immune-challenged fish tested in this study showed increased refuging behaviour 2 days after immune challenge. Increased refuging behaviour may be a strategy that supports both immune and predator defences, as it preserves energetic resources while also reducing the risk of predation and exposure to other pathogens.

Here, fish were tested individually and demonstrated a clear reduction in activity after immune challenge, in line with another similar study [17]. However, this was not the case in previous studies where individuals were tested in the presence of conspecifics. In these studies, immune-challenged fish showed little or no general reduction in activity but demonstrated significant differences in reproductive and social behaviours [21,22]. It has been demonstrated that animals can suppress the expression of sickness behaviours in circumstances where it may be advantageous to do so, such as in reproductive and social contexts [38,53]. Moreover, it is known that animals across taxa often avoid associating with conspecifics showing signs of illness [67–69]. Animals excluded from social groups are generally not able to access the benefits of sociality such as predator defence and enhanced resource availability. Thus, there may be a social cost to the expression of sickness behaviours. Social isolation can itself be a significant source of stress, which is known to trigger neuroinflammation and could thus produce additional effects on behaviour [12,70]. Previous studies in LPS-treated rodents have found that social isolation stress can augment sickness behaviours [71,72]. This is consistent with our findings and raises the question of whether this effect may be driven in part by stress arising from social isolation or by the need to prevent social exclusion. Of course, these mechanisms are not mutually exclusive. If sickness behaviours provide a substantive advantage in recovering from infection but are also accompanied by costs associated with social exclusion then animals may be forced to navigate a trade-off. Additionally, stress may exacerbate the neuroendocrine processes that mediate sickness behaviours. However, the complexity of the interactions between stress, social interaction, immune activity and behaviour means the nexus of these factors requires extensive further investigation.

Broadly, our findings here align with existing studies on the subject of sickness behaviours while also providing new insight into their manifestation over time after an immune challenge. Future avenues of research should combine immunoassays with behavioural analysis in order to examine in detail the immunopathology of the trends observed here. Individual variation in immunocompetence is also known to be an important factor in the physiological and behavioural responses to disease and immune activation. Thus, it would be valuable in future to incorporate measures of immunocompetence, relating them to the magnitude of the immune response and the manifestation of sickness behaviours over time. Finally, as studies on fishes are notably absent from the existing body of research on sickness behaviours it would be beneficial to expand the range of experimental subjects to include more fishes in future analyses.

## Data Availability

All data are available through Dryad [73].

## References

[1] Ashley NT, Wingfield JC. 2011 Sickness behavior in vertebrates. In Ecoimmunology (eds GE Demas, RJ Nelson), pp. 45–91. New York, NY: Oxford University Press.

[2] Ward AJW, Duff AJ, Krause J, Barber I. 2005 Shoaling behaviour of sticklebacks infected with the microsporidian parasite, Glugea anomala. Environ. Biol. Fishes **72**, 155–160. (10.1007/s10641-004-9078-1)

[3] Cray C, Zaias J, Altman NH. 2009 Acute phase response in animals: a review. Comp. Med. **59**, 517–526. 20034426 PMC2798837

[4] Lopes PC, French SS, Woodhams DC, Binning SA. 2021 Sickness behaviors across vertebrate taxa: proximate and ultimate mechanisms. J. Exp. Biol. **224**, jeb225847. (10.1242/jeb.225847) 33942101

[5] Dantzer R. 2001 Cytokine-induced sickness behavior: mechanisms and implications. Ann. N. Y. Acad. Sci. **933**, 222–234. (10.1111/j.1749-6632.2001.tb05827.x) 12000023

[6] Owen-Ashley NT, Turner M, Hahn TP, Wingfield JC. 2006 Hormonal, behavioral, and thermoregulatory responses to bacterial lipopolysaccharide in captive and free-living white-crowned sparrows (Zonotrichia leucophrys gambelii). Horm. Behav. **49**, 15–29. (10.1016/j.yhbeh.2005.04.009) 15967447

[7] Hart BL. 1988 Biological basis of the behavior of sick animals. Neurosci. Biobehav. Rev. **12**, 123–137. (10.1016/s0149-7634(88)80004-6) 3050629

[8] Kushner I, Mackiewicz A. 2020 The acute phase response: an overview. In Acute phase proteins: molecular biology, biochemistry, and clinical applications, pp. 3–19. Boca Raton, FL: CRC Press.

[9] Steel DM, Whitehead AS. 1994 The major acute phase reactants: C-reactive protein, serum amyloid P component and serum amyloid A protein. Immunol. Today **15**, 81–88. (10.1016/0167-5699(94)90138-4) 8155266

[10] Suffredini AF, Fantuzzi G, Badolato R, Oppenheim JJ, O’Grady NP. 1999 New insights into the biology of the acute phase response. J. Clin. Immunol. **19**, 203–214. (10.1023/a:1020563913045) 10471974

[11] Johnson RW. 2002 The concept of sickness behavior: a brief chronological account of four key discoveries. Vet. Immunol. Immunopathol. **87**, 443–450. (10.1016/s0165-2427(02)00069-7) 12072271

[12] Dantzer R, O’Connor JC, Freund GG, Johnson RW, Kelley KW. 2008 From inflammation to sickness and depression: when the immune system subjugates the brain. Nat. Rev. Neurosci. **9**, 46–56. (10.1038/nrn2297) 18073775 PMC2919277

[13] Ots I, Kerimov AB, Ivankina EV, Ilyina TA, Hõrak P. 2001 Immune challenge affects basal metabolic activity in wintering great tits. Proc. R. Soc. Lond. B **268**, 1175–1181. (10.1098/rspb.2001.1636) PMC1088724 11375106

[14] Martin LB, Scheuerlein A, Wikelski M. 2003 Immune activity elevates energy expenditure of house sparrows: a link between direct and indirect costs? Proc. R. Soc. Lond. B **270**, 153–158. (10.1098/rspb.2002.2185) PMC1691219 12590753

[15] Eraud C, Duriez O, Chastel O, Faivre B. 2005 The energetic cost of humoral immunity in the collared dove, Streptopelia decaocto: is the magnitude sufficient to force energy‐based trade‐offs? Funct. Ecol. **19**, 110–118. (10.1111/j.0269-8463.2005.00934.x)

[16] Lopes PC. 2014 When is it socially acceptable to feel sick? Proc. R. Soc. Lond. B **281**, 20140218. (10.1098/rspb.2014.0218) PMC4083780 24943375

[17] Kirsten K, Soares SM, Koakoski G, Carlos Kreutz L, Barcellos LJG. 2018 Characterization of sickness behavior in zebrafish. Brain Behav. Immun. **73**, 596–602. (10.1016/j.bbi.2018.07.004) 29981831

[18] Shakhar K, Shakhar G. 2015 Why do we feel sick when infected—can altruism play a role? PLoS Biol. **13**, e1002276. (10.1371/journal.pbio.1002276) 26474156 PMC4608734

[19] Lochmiller RL, Deerenberg C. 2000 Trade‐offs in evolutionary immunology: just what is the cost of immunity. Oikos **88**, 87–98. (10.1034/j.1600-0706.2000.880110.x)

[20] Mendes-Lima T *et al*. 2020 Prenatal LPS induces sickness behaviour and decreases maternal and predatory behaviours after an LPS challenge. Int. J. Neurosci. **130**, 804–816. (10.1080/00207454.2019.1706505) 31916878

[21] Encel SA, Simpson EK, Schaerf TM, Ward AJW. 2023 Immune challenge affects reproductive behaviour in the guppy (Poecilia reticulata). R. Soc. Open Sci. **10**, 230579. (10.1098/rsos.230579) 37564068 PMC10410201

[22] Encel SA, Schaerf TM, Ward AJW. 2024 Immune challenge changes social behavior in the guppy (Poecilia reticulata). Behav. Ecol. **35**, arad081. (10.1093/beheco/arad081) PMC10410201 37564068

[23] Ferrari MC, Sih A, Chivers DP. 2009 The paradox of risk allocation: a review and prospectus. Anim. Behav. **78**, 579–585. (10.1016/j.anbehav.2009.05.034)

[24] Wilson ADM, Schaerf TM, Ward AJW. 2022 Individual and collective behaviour of fish subject to differing risk-level treatments with a sympatric predator. Behav. Ecol. Sociobiol. **76**, 160. (10.1007/s00265-022-03269-4)

[25] Lima SL, Dill LM. 1990 Behavioral decisions made under the risk of predation: a review and prospectus. Can. J. Zool. **68**, 619–640. (10.1139/z90-092)

[26] Sih A. 1997 To hide or not to hide? Refuge use in a fluctuating environment. Trends Ecol. Evol. **12**, 375–376. (10.1016/s0169-5347(97)87376-4) 21238113

[27] Krause J, Loader SP, McDermott J, Ruxton GD. 1998 Refuge use by fish as a function of body length-related metabolic expenditure and predation risks. Proc. R. Soc. Lond. B **265**, 2373–2379. (10.1098/rspb.1998.0586)

[28] Quinn JL, Cole EF, Bates J, Payne RW, Cresswell W. 2012 Personality predicts individual responsiveness to the risks of starvation and predation. Proc. R. Soc. B **279**, 1919–1926. (10.1098/rspb.2011.2227) PMC3311888 22179807

[29] Hansen MJ, Ligocki IY, Zillig KE, Steel AE, Todgham AE, Fangue NA. 2020 Risk-taking and locomotion in foraging threespine sticklebacks (Gasterosteus aculeatus): the effect of nutritional stress is dependent on social context. Behav. Ecol. Sociobiol. (Print) **74**, 12. (10.1007/s00265-019-2795-4)

[30] Adelman JS, Mayer C, Hawley DM. 2017 Infection reduces anti-predator behaviors in house finches. J. Avian Biol. **48**, 519–528. (10.1111/jav.01058) 29242677 PMC5724792

[31] Eraud C, Jacquet A, Faivre B. 2009 Survival cost of an early immune soliciting in nature. Evolution **63**, 1036–1043. (10.1111/j.1558-5646.2008.00540.x) 19055677

[32] Wood HF. 1953 The relationship between the acute phase response and antibody production in the rabbit. II. The stimulation of Cx-reactive protein response by certain adjuvants and the relation of this response to the enhancement of antibody formation. J. Exp. Med. **98**, 321–329. (10.1084/jem.98.4.321) 13096658 PMC2136248

[33] Tizard I. 2008 Sickness behavior, its mechanisms and significance. Anim. Health Res. Rev. **9**, 87–99. (10.1017/S1466252308001448) 18423072

[34] Kirsten K, Fior D, Kreutz LC, Barcellos LJG. 2018 First description of behavior and immune system relationship in fish. Sci. Rep. **8**, 846. (10.1038/s41598-018-19276-3) 29339805 PMC5770431

[35] Pyke GH. 2005 A review of the biology of Gambusia affinis and G. holbrooki. Rev. Fish Biol. Fisheries **15**, 339–365. (10.1007/s11160-006-6394-x)

[36] Novoa B, Bowman TV, Zon L, Figueras A. 2009 LPS response and tolerance in the zebrafish (Danio rerio). Fish Shellfish Immunol. **26**, 326–331. (10.1016/j.fsi.2008.12.004) 19110060 PMC2748242

[37] Dalmo RA, Kjerstad AA, Arnesen SM, Tobias PS, Bøgwald J. 2000 Bath exposure of Atlantic halibut (Hippoglossus hippoglossus L.) yolk sac larvae to bacterial lipopolysaccharide (LPS): absorption and distribution of the LPS and effect on fish survival. Fish Shellfish Immunol. **10**, 107–128. (10.1006/fsim.1999.0231) 10938728

[38] Gormally BMG, Bridgette K, Emmi A, Schuerman D, Lopes PC. 2022 Female presence does not increase testosterone but still ameliorates sickness behaviours in male Japanese quail. R. Soc. Open Sci. **9**, 220450. (10.1098/rsos.220450) 35620017 PMC9128847

[39] Lopes PC, König B. 2016 Choosing a healthy mate: sexually attractive traits as reliable indicators of current disease status in house mice. Anim. Behav. **111**, 119–126. (10.1016/j.anbehav.2015.10.011)

[40] Moshkin MP, Kondratiuk EI, Gerlinskaia LA. 2009 The sexual behavior, chemosignals and reproductive success in the male mice during activation of nonspecific immune response. Zh. Obshch. Biol. **70**, 515–526. 20063773

[41] Pepels PPLM, Bonga SEW, Balm PHM. 2004 Bacterial lipopolysaccharide (LPS) modulates corticotropin-releasing hormone (CRH) content and release in the brain of juvenile and adult tilapia (Oreochromis mossambicus; Teleostei). J. Exp. Biol. **207**, 4479–4488. (10.1242/jeb.01316) 15557033

[42] Walter T, Couzin ID. 2021 TRex, a fast multi-animal tracking system with markerless identification, and 2D estimation of posture and visual fields. eLife **10**, e64000. (10.7554/eLife.64000) 33634789 PMC8096434

[43] R Core Team. 2022 R: a language and environment for statistical computing. Vienna, Austria: R Foundation for Statistical Computing.

[44] Bates D, Mächler M, Bolker B, Walker S. 2015 Fitting linear mixed-effects models using lme4. J. Stat. Softw. **67**, 1–48. (10.18637/jss.v067.i01)

[45] Fox J, Weisberg S. 2018 An R companion to applied regression. Los Angeles, CA: Sage Publications.

[46] Wickham H. 2016 Ggplot2: elegant graphics for data analysis. New York, NY: Springer-Verlag.

[47] Bonneaud C, Wilson RS, Seebacher F. 2016 Immune-challenged fish up-regulate their metabolic scope to support locomotion. PLoS ONE **11**, e0166028. (10.1371/journal.pone.0166028) 27851769 PMC5113038

[48] Hellenbrand DJ, Quinn CM, Piper ZJ, Morehouse CN, Fixel JA, Hanna AS. 2021 Inflammation after spinal cord injury: a review of the critical timeline of signaling cues and cellular infiltration. J. Neuroinflammation **18**, 284. (10.1186/s12974-021-02337-2) 34876174 PMC8653609

[49] Lopes PC. 2016 LPS and neuroinflammation: a matter of timing. Inflammopharmacology **24**, 291–293. (10.1007/s10787-016-0283-2) 27645902

[50] Swain P, Nayak SK, Nanda PK, Dash S. 2008 Biological effects of bacterial lipopolysaccharide (endotoxin) in fish: a review. Fish Shellfish Immunol. **25**, 191–201. (10.1016/j.fsi.2008.04.009) 18603445

[51] Burness G, Armstrong C, Fee T, Tilman-Schindel E. 2010 Is there an energetic-based trade-off between thermoregulation and the acute phase response in zebra finches? J. Exp. Biol. **213**, 1386–1394. (10.1242/jeb.027011) 20348351

[52] Bonneaud C, Mazuc J, Gonzalez G, Haussy C, Chastel O, Faivre B, Sorci G. 2003 Assessing the cost of mounting an immune response. Am. Nat. **161**, 367–379. (10.1086/346134) 12703483

[53] Lopes PC, Adelman J, Wingfield JC, Bentley GE. 2012 Social context modulates sickness behavior. Behav. Ecol. Sociobiol. **66**, 1421–1428. (10.1007/s00265-012-1397-1)

[54] Moreno‐Rueda G. 2011 Trade‐off between immune response and body mass in wintering house sparrows (Passer domesticus). Ecol. Res. **26**, 943–947. (10.1007/s11284-011-0848-x)

[55] Uller T, Isaksson C, Olsson M. 2006 Immune challenge reduces reproductive output and growth in a lizard. Funct. Ecol. **20**, 873–879. (10.1111/j.1365-2435.2006.01163.x)

[56] Dantzer R. 2001 Cytokine-induced sickness behavior: where do we stand? Brain Behav. Immun. **15**, 7–24. (10.1006/brbi.2000.0613) 11259077

[57] Zamora-Camacho FJ, Reguera S, Rubiño-Hispán MV, Moreno-Rueda G. 2015 Eliciting an immune response reduces sprint speed in a lizard. Behav. Ecol. **26**, 115–120. (10.1093/beheco/aru170)

[58] Ezenwa VO, Stefan Ekernas L, Creel S. 2012 Unravelling complex associations between testosterone and parasite infection in the wild. Funct. Ecol. **26**, 123–133. (10.1111/j.1365-2435.2011.01919.x)

[59] Biro PA, Garland T, Beckmann C, Ujvari B, Thomas F, Post JR. 2018 Metabolic scope as a proximate constraint on individual behavioral variation: effects on personality, plasticity, and predictability. Am. Nat. **192**, 142–154. (10.1086/697963) 30016170

[60] Zamora-Camacho FJ, Comas M, Moreno-Rueda G. 2020 Immune challenge does not impair short-distance escape speed in a newt. Anim. Behav. **167**, 101–109. (10.1016/j.anbehav.2020.07.004)

[61] Stearns SC. 1989 Trade-offs in life-history evolution. Funct. Ecol. **3**, 259. (10.2307/2389364)

[62] Genovart M, Negre N, Tavecchia G, Bistuer A, Parpal L, Oro D. 2010 The young, the weak and the sick: evidence of natural selection by predation. PLoS ONE **5**, e9774. (10.1371/journal.pone.0009774) 20333305 PMC2841644

[63] Møller AP, Nielsen JT. 2007 Malaria and risk of predation: a comparative study of birds. Ecology **88**, 871–881. (10.1890/06-0747) 17536704

[64] Møller AP, Erritzøe J. 2000 Predation against birds with low immunocompetence. Oecologia **122**, 500–504. (10.1007/s004420050972) 28308342

[65] Otti O, Gantenbein-Ritter I, Jacot A, Brinkhof MWG. 2012 Immune response increases predation risk. Evolution **66**, 732–739. (10.1111/j.1558-5646.2011.01506.x) 22380436

[66] Navarro C, Lope F, Marzal A, Møller AP. 2004 Predation risk, host immune response, and parasitism. Behav. Ecol. **15**, 629–635. (10.1093/beheco/arh054)

[67] Kiesecker JM, Skelly DK, Beard KH, Preisser E. 1999 Behavioral reduction of infection risk. Proc. Natl Acad. Sci. USA **96**, 9165–9168. (10.1073/pnas.96.16.9165) 10430913 PMC17750

[68] Behringer DC, Butler MJ, Shields JD. 2006 Avoidance of disease by social lobsters. Nature **441**, 421–421. (10.1038/441421a) 16724051

[69] Zylberberg M, Klasing KC, Hahn TP. 2013 House finches (Carpodacus mexicanus) balance investment in behavioural and immunological defences against pathogens. Biol. Lett. **9**, 20120856. (10.1098/rsbl.2012.0856) 23134781 PMC3565497

[70] Mumtaz F, Khan MI, Zubair M, Dehpour AR. 2018 Neurobiology and consequences of social isolation stress in animal model—a comprehensive review. Biomed. Pharmacother. **105**, 1205–1222. (10.1016/j.biopha.2018.05.086) 30021357

[71] Russell B, Hrelja KM, Adams WK, Zeeb FD, Taves MD, Kaur S, Soma KK, Winstanley CA. 2022 Differential effects of lipopolysaccharide on cognition, corticosterone and cytokines in socially-housed vs isolated male rats. Behav. Brain Res. **433**, 114000. (10.1016/j.bbr.2022.114000) 35817135

[72] Yee JR, Prendergast BJ. 2010 Sex-specific social regulation of inflammatory responses and sickness behaviors. Brain Behav. Immun. **24**, 942–951. (10.1016/j.bbi.2010.03.006) 20303405 PMC2897937

[73] Encel S, Ward A. 2024. Immune challenge affects risk sensitivity and locomotion in mosquitofish (Gambusia holbrooki) [Dataset]. Dryad Digital Repository. (10.5061/dryad.xsj3tx9q8) PMC11521614 39479234

